# The heterogeneous well-being effects of intergenerational mobility perceptions

**DOI:** 10.1177/13591053231187345

**Published:** 2023-07-19

**Authors:** Alexi Gugushvili

**Affiliations:** University of Oslo, Norway

**Keywords:** Georgia, health, perceptions, social mobility, treatment effect estimators, well-being

## Abstract

Individuals make comparisons with their parents which determine their intergenerational mobility perceptions, yet very little is known about the areas used for intergenerational comparison and whether these matter for individuals’ well-being. In 2021 we commissioned a nationally representative survey in Georgia in which we explicitly asked 1159 individuals an open-ended question on the most important areas in their intergenerational comparisons. More than 170 types of answers were provided by respondents and many of these responses went beyond the standard indicators of intergenerational mobility. We show that the areas of intergenerational comparison significantly differ between those who perceive themselves as being downwardly and upwardly mobile or immobile using the measure of mobility previously validated in cross-national research. Using, among other statistical approaches, treatment effects estimators, we demonstrate that some areas of intergenerational comparison, particularly in terms of income attainment, are significantly and consistently associated with internationally validated measures of well-being.

## Introduction

Researchers across social science disciplines have recently paid more attention to perceived aspects of socioeconomic position (SEP) and their implications for various life outcomes such as health and well-being (Richards et al., 2023; Schneider, 2019). Many of these studies find that, even after objective aspects of SEP such as education, occupation, and income are accounted for, individuals’ perceived position in the social hierarchy is associated with their well-being outcomes. Scholars usually assume that perceived SEP involves some form of social comparison because individuals have to size themselves up against others to learn about their place in the socioeconomic structure (Alexander et al., 2021; Hoebel and Lampert, 2020).

Social mobility scholars, in turn, have started to explore the trends, causes, and consequences of intergenerational mobility perceptions. Intergenerational mobility usually refers to a change in the SEP of offspring in relation to their parents. This change can be either attaining a higher SEP than the previous generation (upward mobility) or moving to a lower SEP than the previous generation (downward mobility). One of the main findings of this emerging scholarship is that there are major differences between objective trends in social mobility, as defined by the conventional measures of SEP such as education, occupation, and income, and changes in perceived intergenerational mobility (Berger and Engzell, 2020). In terms of the factors influencing why individuals perceive themselves as being upwardly or downwardly mobile, overall economic development across individuals’ life course, subjective social position, household income, and the information about equality of opportunity that individuals possess are one of the most important explanations (Gugushvili, 2021c, 2022; Gugushvili and Zelinska, 2023; Kelley and Kelley, 2009).

Understanding the consequences of perceived social mobility on individuals’ well-being can build on the long-standing tradition in sociology and social psychology that explores various implications of intergenerational mobility based on objective measures of SEP (Day and Fiske, 2019; Lopreato, 1967). This scholarship has so far resulted in mixed findings partially determined by the methodological challenges related to collinearity between origin SEP and destination SEP and mobility between these two in conventional regression models (Bulczak et al., 2022). Despite recent proposals which mitigate the described statistical concerns (Luo, 2022), the problem of collinearity can be addressed by relying on individuals’ intergenerational perceptions, since this type of mobility is related to but is not linearly associated with individuals’ objective mobility experiences (Präg and Gugushvili, 2021).

## Framework

### Intergenerational mobility perceptions and well-being

Different theories predict that intergenerational mobility affects individuals’ well-being because they might feel disconnected and disappointed with their new social environment after experiencing intergenerational mobility (Newman, 1999). On the other hand, individuals might be empowered and feel they have greater control over their lives after experiencing intergenerational upward mobility, the process which is described in the “rising from rags” hypothesis (Gugushvili et al., 2019b). Additionally, the so-called acculturation thesis claims that rather than social mobility, it is the modes of socialization, practices, and environments at the origin and destination positions that determine individual well-being outcomes (Blau, 1956). Importantly, these theoretical approaches explicitly assume that individuals need to be aware of their mobility experiences and make an intergenerational comparison between their current and past SEP.

Recent empirical results on the implications of perceived social mobility have produced more consistent findings than is the case for research on the consequences of objective intergenerational mobility. For instance, a study of Russian data suggests that perceived social mobility is strongly associated with both mental and physical health as measured by the Short-Form-12 Health Survey (Gugushvili and Präg, 2021). The studies of German and Polish respondents show that perceived social mobility is associated with, respectively, life satisfaction and self-rated health (Gugushvili et al., 2022; Präg and Gugushvili, 2021). The latter findings are in line with those of a South Korean study which shows that life satisfaction is significantly linked to individuals’ perception of doing better than their parents did (Hsiao et al., 2020).

The described research is informative and suggests that there are significant links between perceived social mobility and various measures of well-being, but it does not explore the heterogeneous well-being effects of intergenerational mobility perceptions. To further investigate the implications of intergenerational mobility perceptions for well-being, it is important to understand what intergenerational comparison implies for individuals (Gugushvili, 2021b).

### Why should we expect heterogenous well-being effects?

It is largely unknown which factors are most important for individuals when they compare their position in life to that of their parents. Existing social surveys which include information about perceived intergenerational mobility only ask individuals if they feel that they are downwardly or upwardly mobile or immobile. This approach is informative but it cannot explain which specific aspects of intergenerational comparison are most important for individuals and whether or not these specific areas of comparison have any implications for individuals’ well-being. For instance, the survey questions on perceived social mobility in the International Social Survey Programme (ISSP, 2012) and the United States General Social Survey (Berger and Engzell, 2020) explicitly ask respondents to make an intergenerational comparison in terms of jobs and standard of living but, to our knowledge, no studies exist which would indicate whether or not these are the most important areas of life by which mobile individuals compare themselves with their parents.

If we assume that individuals significantly differ by the areas according to which they make the intergenerational comparison, should we also expect that certain comparisons will be more strongly related to individuals’ well-being than others? Existing research using objective measures of social mobility suggests that different operationalization of SEP significantly affects the outcomes of the analysis, as various measures such as education, occupation, or income are linked to well-being via different channels such as, respectively, health-related knowledge, beneficial social connections, and material resources for better life conditions (Mackenbach, 2019). Likewise, individuals might assign different weights to different aspects of life in intergenerational comparisons, with some being more relevant for well-being than others. From related streams of research, we know that some aspects of social comparison are associated with worse or better health outcomes. For instance, in a study of post-communist countries individuals who compared themselves with their parents and their own families before the start of the transition were less likely to report good health compared with those who did not compare their economic situation with any specific reference group (Gugushvili et al., 2019a).

Questionnaires used in social surveys do not allow individuals to describe specific areas of comparison which they refer to when forming their intergenerational mobility perceptions. On the other hand, some studies using convenience sampling explicitly investigate the nature of perceived intergenerational mobility but their findings are not generalizable to an entire society or other countries (Duru-Bellat and Kieffer, 2008). To fill this gap, in January 2021, we commissioned a nationally representative survey, the first of its kind, in the post-communist society of Georgia in which we asked participants an open-ended question about the most important areas in their intergenerational comparisons. The survey also collected information on individuals’ physical and mental health, life satisfaction and various social origin, sociodemographic and socioeconomic characteristics. The latter allows us to investigate the areas of intergenerational comparison in terms of perceptions about mobility and immobility and whether or not these specific areas of intergenerational comparison have implications for individuals’ well-being.

## Research design

### Dataset

In this study, we use data from a nationally representative survey that we commissioned from the Caucasus Research Resource Center in the country of Georgia. Data collection took place in January 2021 and the survey’s coverage was the country’s adult population. The data were collected via an interviewer-administered phone survey based on a sample design generated by the random digit dialing (RDD) survey approach. The survey’s response rate according to the American Association for Public Opinion Research’s (AAPOR) RR1 was 29%. Individuals were the primary sampling units and each phone number was treated as a personal-use device. The sample strata were based on the capital city, urban, and rural settlements. Every 10th interview was checked via call-backs and the average theoretical margin of error was 2.7%. This study complies with the local legislation and institutional requirements and was conducted in adherence to all AAPOR ethical research standards. After the listwise deletion of observations with missing information, 1159 out of 1270 individuals were available for our multivariable analysis. For robustness check, we also imputed missing data using multiple imputations via the MICE package in Stata, version 17, allowing for 10 sets of multiple imputations and combining them using Rubin’s (1987) rules. The results, presented in Supplemental Materials, Table S1, are identical to the ones with the listwise deletion reported in the main analysis. The dataset for the replication of the present study is freely available via Open Science Framework (Gugushvili, 2021a).

### Perceived social mobility and areas of intergenerational comparison

For deriving information about perceived intergenerational mobility, the following statement was read to the participants of our survey: “I have done worse in life than my parents when they were of my age.” The 5-point Likert scale answer options varied from “completely disagree” to “completely agree.” This measure of perceived intergenerational mobility has been previously validated in comparative research across a large number of European societies (Djankov et al., 2016; Gugushvili, 2016, 2019). We classified these answers into five categories of perceived intergenerational mobility: “completely agree” = strong downward mobility (6.5% of answers), “agree” = downward mobility (34.5%), “neither agree nor disagree” = immobility (20.7%), “disagree” = upward mobility (31.7%), and “completely disagree” = strong upward mobility (6.7%) (Gugushvili, 2016).

The perceived intergenerational mobility question was followed by an open-ended question: “What is the most important factor in comparing yourself to your parents?” Interviewees were instructed to write down the exact answers made by the respondents. If long responses were given, interviewees were further asked to specify what was the most important area of their intergenerational comparison. Overall, in addition to stating “don’t know” or not making any intergenerational comparison, 17.8% of the sample, 173 different types of responses were provided to the interviewers. We manually examined individuals’ responses and classified them together into seven major groups when the answers were semantically related to each other by similar words or the context of responses. The full list of these answers and codes for their grouping are shown in the Supplemental Materials, Table S2.

### Well-being outcomes

We consider well-being as a multifaceted construct and use three self-reported outcome measures that, in line with the World Health Organization’s (WHO) definition of well-being, reflect not just the physical health of individuals but also their psychological well-being. The survey collected information on respondents’ self-rated physical and mental health by asking two separate questions: “How would you assess your physical health?” and “How would you assess your mental health?” with the following answer options for both health measures: “very bad” (1.8% for physical health and 0.6% for mental health), “bad” (11.4% and 2.5%), “fair” (36.9% and 19.9%), “good” (38.4% and 54.5%), and “very good” (11.4% and 22.1%) (Pinillos-Franco and Kawachi, 2022). The question on life satisfaction included in the survey had the following form: “How satisfied are you with your life as a whole today?” with answer options varying from “very dissatisfied” = 0 to “very satisfied” = 10 (mean = 5.7, SD = 2.3) (Fors Connolly and Gärling, 2022). Correlation coefficients between the described well-being outcomes, reported in the Supplemental Materials, Table S3, suggest that physical and mental health are moderately related to each other, but the association between health and life satisfaction is weak.

### Covariates of well-being

When analyzing the implications of specific areas of intergenerational comparison, we need to account for other known predictors of well-being which might also be associated with the modes of comparison that individuals use. Age and gender are basic covariates of well-being. Parental and own educational attainment are both operationalized with primary, secondary, vocational, and tertiary education and are known to be important predictors of health and well-being. Household income, especially among economists, is considered as one of the most important determinants of life satisfaction (Veenstra and Vanzella-Yang, 2020). The variable which we use in this study is derived from the absolute levels of declared monthly household income which is then transformed into tertiles of income.

From different labor market characteristics, we consider the following types of attachment which are known to be linked with individuals’ well-being (Krug and Eberl, 2018): employed, self-employed, retired, and all other labor market categories combined. Geographic differences in economic development and unequal access to healthcare are important determinants of well-being in Georgia (Hagan et al., 2019). We divided individuals’ types of settlement by residency in an urban area, a small town, a rural area, and the capital city. Internally displaced individuals (IDP) are known to be disadvantaged in Georgia and we create a separate dummy variable for them (Singh et al., 2018). Lastly, we account for the day of the interview fixed effects as the survey was conducted during the Covid-19 related restrictions, and some daily changes in infection rates and regulations could have affected individuals’ responses and well-being outcomes. The descriptive statistics for and correlation coefficients of the covariates of well-being are shown in the Supplemental Materials, Tables S3 and S4, which suggests that the considered variables are not strongly associated with each other.

### Statistical analyses

We first present the descriptive statistics for the most important areas of intergenerational comparison and relate them to the perceptions of downward and upward mobility and immobility using a chi-square test and multinomial logistic regression. To understand if there are links between mobility perceptions and well-being outcomes, and if there are heterogeneous effects due to the areas of intergenerational comparison, we consecutively fit unadjusted and adjusted linear regression models (OLS) for upward and downward mobility perceptions, with the perception of immobility being the reference category. Following previous research, we use the full range of ordinal answer options for self-rated physical and mental health and treat them as continuous measures (McClure et al., 2019). We use the same OLS approach for life satisfaction outcome as this variable resembles normal distribution (see Figure S1 in the Supplemental Materials). Next, we fit identical models but substitute downward and upward mobility perceptions with a specific area of comparison to identify if there are heterogeneous well-being effects among individuals who perceive themselves as being intergenerationally mobile. We aggregate strong upward (strong downward) and upward (downward) mobility perceptions due to low numbers of observations per area of intergenerational comparison.

To assess the validity of the results derived from conventional regressions, we use treatment effects estimators via regression adjustment of the OLS models (Negi and Wooldridge, 2021). The advantages of using treatment effects estimators are that they mimic randomized experiments and have no strict assumptions about the functional form of an association between treatment and outcome. Regression adjustment using the Stata command “teffects ra” is based on a two-step approach to estimating treatment effects. First, we fit separate regression models of the outcome (physical health, mental health, and life satisfaction) for a set of covariates for each considered area of intergenerational comparison. Second, we compute the averages of the predicted outcomes for each comparison area. The contrasts among the averages for the specific type of comparison versus making no comparison provide the estimates of average treatment effects (ATE). In addition to reporting the standard ATE estimates, we also calculate ATE as a percentage of the mean value of well-being outcomes for those who perceive themselves as being intergenerationally immobile. We only estimate treatment effects for those areas of intergenerational comparison which are significantly associated with well-being in the conventional analysis.

## Results

### What are the most important areas of intergenerational comparison?

We start by describing the Treemap of the most salient areas of comparison which individuals mentioned when asked to name the factors by which they form intergenerational mobility perceptions. In Figure S2 in the Supplemental Materials, we see that in addition to stating “don’t know” or not making any intergenerational comparison, the largest single area of comparison, around 14% of answers, is individuals’ educational attainment. This area of comparison also includes 4.3% of respondents who mentioned education as the first factor along with one other factor such as employment or self-realization. Income is the second largest area of intergenerational comparison with around 13% of responses. The career and occupational category accounts for around 12% of responses, relating to occupational attainment, labor market success, and other job-related characteristics. About one-tenth of respondents mentioned factors associated with family, friends, and relationships as the most important areas of life which they use to compare with their parents.

We aggregate economic conditions, around 8% of the responses, as a separate comparison area that covers the changing economic situation around individuals as well as the levels of poverty. A related yet distinct area of intergenerational comparison is the category of living conditions, with 8% of responses, in which the most salient aspect was individuals’ housing. We have combined all of the remaining answers into the “other” comparison category, yet this label does not reflect the diversity of answers that individuals provide. Among other areas of life, respondents mentioned religiosity, freedom to conduct business, independence, honesty, happiness, having new possibilities, not living in the Soviet Union, intellectual abilities, lifestyles, peace, self-realization, and being an IDP as the most important factors in their intergenerational comparisons.

### Do intergenerational mobility perceptions vary by areas of comparison?

**Figure 1.**
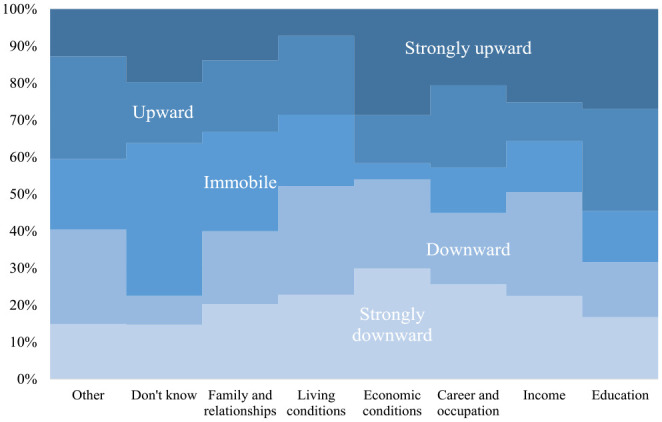
Areas of intergenerational comparison by individuals’ mobility perceptions. Source: Own calculations based on the Perceived Social Mobility dataset. χ^2^ test = 173.25, *p*-value = 0.000.

Figure 1 shows how the share of perceptions of intergenerational downward and upward mobility and immobility varies by specific areas of comparison. The chi-square test suggests that differences in mobility perceptions are significant across the areas of comparison (exact distributions are shown in Table S5 in the Supplemental Materials). A few characteristics stand out in the visualized distribution. Strong upward mobility perceptions are prevalent among those who make intergenerational comparisons by economic conditions, education, and income. If we jointly consider upward and strongly upward mobility perceptions, then the highest share of individuals reporting being upwardly mobile are among those who make the intergenerational comparison by educational attainment. Immobility perceptions are particularly salient among those who do not know what their area of comparison is, or who explicitly state that they do not compare with their parents. Downward mobility perceptions are highest among those who compare with their parents by income, economic, and living conditions. On the other hand, downward mobility perceptions are also lowest among those who make intergenerational comparisons by educational attainment, do not know what factors they use for comparison, or do not compare at all with their parents.

In the Supplemental Materials, Table S6, we fit a multinomial logistic model to see if the results shown in Figure 1 are affected by individuals’ basic sociodemographic characteristics such as age and gender. The derived results are essentially identical to those shown in Figure 1 suggesting that, for instance, those who stress education as the most important area of comparison are more likely to perceive themselves as being upwardly mobile, while those who stress economic conditions as the main area of intergenerational comparison are more likely to perceive themselves as being downwardly mobile.

### Do intergenerational mobility perceptions matter for well-being?

We now start inquiring as to whether or not different areas of comparison play a role in a potential association between perceived intergenerational mobility and individuals’ well-being. In Table S7 of the Supplemental Materials, we fit OLS models for physical and mental health and life satisfaction outcomes to see if perceived intergenerational mobility is associated with our dependent variables. We checked the central regression assumptions such as linearity, multivariate normality, and homoscedasticity (variance inflation factors (VIFs) for all the variables are below 5 as shown in the Supplemental Materials, Table S8). Although the results in Models 1 and 3 in Table S7 suggest that those who perceive themselves as being upwardly mobile have better physical and mental health, these associations become insignificant when we include other variables in Models 2 and 4 (the results are identical using ordered probit models in Table S9). Yet, downward and upward mobility perceptions are associated with, respectively, lower and higher life satisfaction in Models 5 and 6. Full regression results are shown in the Supplemental Materials, Table S10.

### Areas of intergenerational comparison, well-being, and treatment effects estimates

**Figure 2 .**
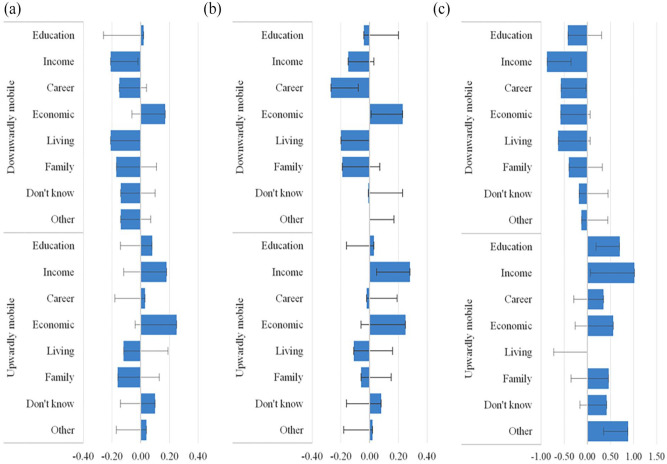
Areas of intergenerational comparison among individuals with downward and upward mobile perceptions and wellbeing outcomes: (a) physical health, (b) mental health, and (c) life satisfaction. Source: Own calculations based on the Perceived Social Mobility dataset. All models account for age, age squared, gender, interview day fixed effects, parental education, respondents’ education, household income, labor market status, type of settlement, IDP status. For presentational purpose, the lines depict 95% confidence estimates toward the reference estimate of zero.

In Figure 2, we investigate whether specific areas of intergenerational comparison among individuals who have downward and upward mobility perceptions are associated with well-being, with individuals who express immobility perceptions being in the reference category. We fit identical regressions, as in Models 2, 4, and 6 in Table S7, but this time we disaggregate mobility perceptions by areas according to which individuals compare their life situation to that of their parents. The results suggest that there is variation in well-being effects among the comparison areas used by downwardly and upwardly mobile individuals (full regression results are shown in the Supplemental Materials, Tables S11 and S12). For self-rated physical health, individuals with income as the main area of intergenerational comparison have a 0.21 (β −0.21, 95% CI −0.40, −0.02) points lower score. For self-rated mental health, we did not observe any effects of mobility perceptions in Model 4, Table S10, but Figure 2(b) suggests that intergenerational comparison by income among individuals with upward mobility perceptions has a positive association with mental health (β 0.28, 95% CI 0.06, 0.51). In addition, mental health is worse among those who perceive themselves as being downwardly mobile and make the intergenerational comparison by career and occupational outcomes (β −0.27, 95% CI −0.46, −0.07).

Among individuals with downward mobility perceptions, we also see a negative association between comparisons based on income and the career and occupation categories, on the one hand, and life satisfaction, on the other hand. Life satisfaction is also higher among individuals with upward mobility perceptions who make intergenerational comparisons by education and income attainment. We also find that comparing with one’s parents by factors that are not included in the main areas of comparison is related to greater life satisfaction in Figure 2(c) (β 0.88, 95% CI 0.34, 1.41).

Finally, in Table 1 we present results for treatment estimators using regression adjustment for those areas of intergenerational comparison which demonstrate statistically significant associations with well-being outcomes in conventional models, as shown in Figure 2. These treatment estimators are informative as they are a more robust test of the detected associations than the conventional OLS regressions. We do not find that intergenerational downward income comparison is linked to individuals’ physical health, but comparing with one’s parents by career and occupation is significantly and negatively associated with mental health among individuals with downward mobility perceptions. Those who compare with their parents by career have 0.16 points lower mental health than individuals with perceptions of immobility. Significant effects for the described two areas of intergenerational comparison are also observed for life satisfaction among individuals with downward mobility perceptions. Further, for the latter measure of individuals’ well-being, comparison by income (40% better score than immobile individuals, 95% CI 8%, 71%) and by other factors (18% better score than immobile individuals, 95% CI 9%, 28%) are also positively associated with life satisfaction among those who perceive themselves as being upwardly mobile.

**Table 1.** Average treatment effects (ATE) estimates from regression adjustment treatment estimators.

	ATE estimates	ATE as % of well-being outcomes among individuals with immobility perceptions
*Physical health*		
Downward: Income comparison	–0.14 [–0.36, 0.07]	–4.1 [–10.3, 2.1]
*Mental health*		
Downward: Career comparison	–0.61 [–0.78, –0.43]	–15.4 [–19.8, –11.1]
Upward: Income comparison	–0.05 [–0.16, 0.05]	–2.4 [–11.1, 6.4]
*Life satisfaction*		
Downward: Income comparison	–1.11 [–2.08, –0.14]	–19.2 [–35.8, –2.58]
Downward: Career comparison	–0.70 [–1.20, –0.20]	–12.1 [–20.5, –3.64]
Upward: Education comparison	0.24 [–0.49, 0.98]	4.15 [–8.62, 16.9]
Upward: Income comparison	2.29 [0.50, 4.08]	39.6 [8.37, 70.8]
Upward: Other	1.06 [0.54, 1.58]	18.3 [8.93, 27.7]

Source: Own calculations based on Perceived Social Mobility dataset.

95% confidence intervals in brackets. Outcome independent variables include age, age squared, gender, parental education, respondents’ education, household income, labor market status, type of settlement, IDP status.

## Discussion

This study contributes to the emerging literature on perceived intergenerational mobility and its effects on well-being. Inconclusive findings in research on the consequences of objective social mobility on individuals’ well-being have motivated researchers to explore various implications of perceived social mobility. The existing evidence, though, stems exclusively from the analyses of social surveys in which participants with intergenerational mobility perceptions are treated as a homogenous group of individuals. We know from the sociology and social psychology literature that individuals differ by the types of social comparisons they make—comparing with themselves in the past or with others who are higher or lower in the social hierarchy—and these comparisons matter for various aspects of individuals’ well-being (Wolff et al., 2010). Yet, there is virtually no research on the types of comparisons which individuals make when forming their intergenerational mobility perceptions. Therefore, our goal in this study was to contribute to the emerging literature by identifying the most important areas of intergenerational comparison and analyzing if these areas were linked to individuals’ well-being.

In the nationally representative survey conducted in Georgia, we explicitly asked individuals which areas of life they compared with their parents while forming intergenerational mobility perceptions. Apart from the typically high share of non-responses with open-ended questions (Meitinger and Kunz, 2022), we have aggregated more than 170 types of answers into main areas of intergenerational comparison and have established that factors such as education, income, occupation, and economic and living conditions are most important when individuals form their mobility perceptions. It is not surprising that a significant share of individuals, especially among those who perceived being upwardly mobile, chose education as the main comparison factor because Georgia, among many countries that belonged to the socialist block, experienced a major educational expansion since around the mid-20th century (Bernardi and Ballarino, 2016). Educational expansion might lead to upward mobility perceptions which are also objectively true. Overall, the described areas of intergenerational comparison, such as educational, occupational, and income attainment, are intensively researched by sociologists and economists. Nonetheless, one of the main findings from our study with the potential theoretical implication is that, in addition to the standard measures of SEP, a significant share of individuals does not know their comparison factor, or compare themselves with their parents according to factors that are not classified in the standard measures, or even make the intergenerational comparison based on family relationships and social aspects of life.

We further explore if specific areas of intergenerational comparison are associated with individuals’ well-being outcomes. We first fit models of general downward and upward intergenerational mobility perceptions to understand how these perceptions are linked to individuals’ self-rated physical and mental health and life satisfaction. In models with an array of social origin, sociodemographic and socioeconomic variables, we do not find that perceived intergenerational mobility is associated with health outcomes, but that both downward and upward mobility perceptions are significantly linked with, respectively, lower and higher life satisfaction. Our main goal has been to identify heterogeneous effects among individuals with different mobility perceptions and this is why we substitute upward and downward mobility variables with specific areas of intergenerational comparisons. The latter approach confirms that certain areas of comparison have significant associations with well-being outcomes. This finding implies that previous research that has aggregated the general perceptions of intergenerational mobility has also combined individuals with different patterns of intergenerational comparison, which makes it difficult to observe heterogenous well-being effects in the conventional analysis of perceived social mobility.

The most consistent association which we identify is that of intergenerational comparison by income among downwardly mobile individuals being linked to worse self-rated health and lower life satisfaction. Further, among upwardly mobile individuals’ intergenerational income comparison is also associated with better mental health and greater life satisfaction. These findings can be linked with some recent studies which suggest that income is more strongly associated with individuals’ health than other aspects of SEP (Hoffmann et al., 2019). As for research on the implications of intergenerational income mobility for well-being, an ecological study for the United States has found a positive link between country-level income mobility and life expectancy (Venkataramani et al., 2020). Furthermore, the importance of perceived mobility in income may be particularly relevant in Georgia which has suffered major economic and political turmoil since the end of the 1980s (Stolz et al., 2023).

We have also identified that intergenerational comparison in career and occupational attainment is linked to worse mental health and lower life satisfaction among individuals with downward mobility perceptions. The theoretical significance of this finding is that occupational mobility is the central area of inquiry in sociological research on social mobility. It is believed that occupation-based social class is a more robust and long-term indicator of individuals’ material well-being, job security, and unemployment risk (Galobardes, 2006). The transition period in Georgia, along with other post-communist countries, was associated with deindustrialization and a major reshuffling of the occupational structure which has likely affected individuals’ perceptions of intergenerational occupational mobility. Further, one of the main theoretical implications of our study is that a group of other factors for comparing intergenerational comparison is significantly linked to the life satisfaction of upwardly mobile individuals. This aggregated area of intergenerational comparison includes, among others, factors such as freedom, religiosity, new opportunities, and lifestyles that post-communist generations are more likely to enjoy than the previous generations (Guriev and Melnikov, 2018).

### Limitations

This study has its limitations. First, although we control for an array of important covariates of well-being and also use treatment effects estimators via regression adjustment, we are not able to identify causal associations in our observational data. Second, the sample size of the survey does not allow us to have sufficiently large groups for certain areas of intergenerational comparison to pinpoint their potential effect on individuals’ well-being. Third, the main areas of intergenerational comparison such as career, income, economic conditions, living conditions, and housing are closely related to each other, and this can potentially cause the multicollinearity problem in predicting well-being outcomes. Fourth, our measure of health and well-being is based on individuals’ reports which are important predictors of actual morbidity and mortality (Lorem et al., 2020), but having objectively measured outcome variables could improve the validity of our findings. Fifth, the survey was conducted during the Covid-19 pandemic which may have affected both individuals’ well-being and the areas on which they have based their intergenerational comparisons. Sixth, our findings may be affected by the issue of health selection which means that individuals with downward and upward mobility perceptions had, respectively, worse or better well-being outcomes even before forming their mobility perceptions (Bulczak and Gugushvili, 2022; Hoffmann et al., 2018).

## Conclusion

Notwithstanding its shortcomings, the main conclusion of our study and its implication for theory is that individuals significantly differ by area of comparison when they perceive themselves as being intergenerationally upwardly or downwardly mobile or immobile. While many compare themselves with their parents by standard measures of educational, occupational, and income attainment, other areas such as living conditions, broader economic development, social relationships, and other aspects of life represent a substantial share of overall intergenerational comparisons. These under-explored areas of intergenerational comparison deserve further attention from social scientists. Future surveys concerned with both objective and subjective aspects of social mobility might consider including questions related to aspects of life that go beyond individuals’ SEP. Georgia, as a transitional society, is an interesting case study but it remains to be seen what areas of intergenerational comparison individuals choose to make in Western welfare democracies. As we show in this study, some areas of intergenerational comparison matter more for individuals’ well-being, and this further underscores the importance of differentiating various types of individuals among those who perceive themselves as being intergenerationally mobile in a downward or upward direction.

## Supplemental Material

sj-docx-1-hpq-10.1177_13591053231187345 – Supplemental material for The heterogeneous well-being effects of intergenerational mobility perceptions Supplemental material, sj-docx-1-hpq-10.1177_13591053231187345 for The heterogeneous well-being effects of intergenerational mobility perceptions by Alexi Gugushvili in Journal of Health Psychology

sj-dta-2-hpq-10.1177_13591053231187345 – Supplemental material for The heterogeneous well-being effects of intergenerational mobility perceptions Supplemental material, sj-dta-2-hpq-10.1177_13591053231187345 for The heterogeneous well-being effects of intergenerational mobility perceptions by Alexi Gugushvili in Journal of Health Psychology This article is distributed under the terms of the Creative Commons Attribution 4.0 License ( http://www.creativecommons.org/licenses/by/4.0/) which permits any use, reproduction and distribution of the work without further permission provided the original work is attributed as specified on the SAGE and Open Access pages ( https://us.sagepub.com/en-us/nam/open-access-at-sage).
