# Supplementary material for: The heterogeneous well-being effects of intergenerational mobility perceptions
Source: J Health Psychol. 2023 Jul 19;29(2):99–112. doi: 10.1177/13591053231187345 (PMC11378449; doi:10.1177/13591053231187345)
Supplement: sj-docx-1-hpq-10.1177_13591053231187345 – Supplemental material for The heterogeneous well-being effects of intergenerational mobility perceptions [file sj-docx-1-hpq-10.1177_13591053231187345.docx]

**Supplementary materials**

**Table S1.** Perceived social mobility and wellbeing outcomes with imputed data

|  | *Physical health*  *(1=very bad, very good=5)* | | *Mental health*  *(1=very bad, very good=5)* | | *Life satisfaction*  *(0=dissatisfied, 10=satisfied)* | |
| --- | --- | --- | --- | --- | --- | --- |
|  | Model 1: OLS without controls | Model 2: OLS  with controls | Model 3: OLS without controls | Model 4: OLS  with controls | Model 5: OLS without controls | Model 6: OLS  with controls |
| *Perceived mobility* |  |  |  |  |  |  |
| (Strongly) downward | -0.15* | -0.12 | -0.10 | -0.08 | -0.64*** | -0.51** |
|  | [-0.27,-0.03] | [-0.24,0.00] | [-0.21,0.00] | [-0.20,0.03] | [-0.97,-0.30] | [-0.84,-0.18] |
| Immobile |  |  |  |  |  |  |
| (Strongly) upward | 0.09 | 0.04 | 0.10 | 0.07 | 0.68*** | 0.52** |
|  | [-0.03,0.21] | [-0.08,0.17] | [-0.01,0.20] | [-0.03,0.18] | [0.36,1.00] | [0.21,0.83] |
| Intercept | 4.51*** | 4.25*** | 4.42*** | 4.46*** | 7.09*** | 5.45*** |
|  | [4.27,4.75] | [3.82,4.67] | [4.20,4.63] | [4.09,4.83] | [6.50,7.68] | [4.43,6.48] |
| Number of imputations | 10 | 10 | 10 | 10 | 10 | 10 |
| Observations | 1270 | 1270 | 1270 | 1270 | 1270 | 1270 |
| Average RVI | 0.0217 | 0.0673 | 0.0129 | 0.0747 | 0.0421 | 0.0807 |

*Notes:* * p<0.05, ** p<0.01, *** p<0.001. 95% confidence intervals in brackets. Models with control variables account for for age, age squared, gender, interview day fixed effects, parental education, respondents’ education, household income, labor market status, type of settlement, IDP status. *Source:* Own calculations based on Perceived Social Mobility dataset.

**Table S2:** Full list of aspects of intergenerational comparison

| Selected Group | ID | Respondents’ answers | Number of individuals | Share in the sample |
| --- | --- | --- | --- | --- |
| Don't know | 1 | Don't know | 226 | 17.8 |
| Other aspects | 2 | A little bit of everything | 1 | 0.1 |
| Other aspects | 3 | Ability | 1 | 0.1 |
| Other aspects | 4 | Ability to fight | 1 | 0.1 |
| Other aspects | 5 | Agriculture | 1 | 0.1 |
| Other aspects | 6 | Almost the same | 2 | 0.2 |
| Living conditions | 7 | Are living Better | 2 | 0.2 |
| Other aspects | 8 | At that period, there was everything an | 1 | 0.1 |
| Other aspects | 9 | At that period, they were living in a b | 1 | 0.1 |
| Economic conditions | 10 | At that period, they were not poor and | 1 | 0.1 |
| Other aspects | 11 | Authority | 1 | 0.1 |
| Other aspects | 12 | Being a better man, charity, profession | 1 | 0.1 |
| Living conditions | 13 | Better life/conditions | 10 | 0.8 |
| Other aspects | 14 | Business activities | 1 | 0.1 |
| Family and relationships | 15 | By the number of children | 1 | 0.1 |
| Don't know | 16 | Can't compare | 4 | 0.3 |
| Career | 17 | Career | 89 | 7.0 |
| Career | 18 | Career and Intellect | 1 | 0.1 |
| Career | 19 | Career and finance | 3 | 0.2 |
| Career | 20 | Career and life better conditions | 1 | 0.1 |
| Family and relationships | 21 | Childhood | 1 | 0.1 |
| Family and relationships | 22 | Children | 24 | 1.9 |
| Other aspects | 23 | Church life | 1 | 0.1 |
| Other aspects | 24 | Continued parents' trade | 1 | 0.1 |
| Other aspects | 25 | Demographics | 1 | 0.1 |
| Economic conditions | 26 | Development | 1 | 0.1 |
| Other aspects | 27 | Did their best | 1 | 0.1 |
| Other aspects | 28 | Different period | 24 | 1.9 |
| Don't know | 29 | Don't compare | 7 | 0.6 |
| Economic conditions | 30 | Economic conditions | 95 | 7.5 |
| Economic conditions | 31 | Economy | 1 | 0.1 |
| Education | 32 | Education | 109 | 8.6 |
| Education | 33 | Education and better conditions | 1 | 0.1 |
| Education | 34 | Education and career | 12 | 0.9 |
| Education | 35 | Education and economic status | 1 | 0.1 |
| Education | 36 | Education and employment | 5 | 0.4 |
| Education | 37 | Education and employment were available | 1 | 0.1 |
| Education | 38 | Education and finance | 5 | 0.4 |
| Education | 39 | Education and happiness | 1 | 0.1 |
| Education | 40 | Education and hard working | 1 | 0.1 |
| Education | 41 | Education and health | 1 | 0.1 |
| Education | 42 | Education and income | 9 | 0.7 |
| Education | 43 | Education and interests | 1 | 0.1 |
| Education | 44 | Education and it was a better time | 1 | 0.1 |
| Education | 45 | Education and job experience | 1 | 0.1 |
| Education | 46 | Education and networking | 1 | 0.1 |
| Education | 47 | Education and now there are more opportunities | 1 | 0.1 |
| Education | 48 | Education and personal relationship | 1 | 0.1 |
| Education | 49 | Education and principality | 1 | 0.1 |
| Education | 50 | Education and privileges | 1 | 0.1 |
| Education | 51 | Education and property | 1 | 0.1 |
| Education | 52 | Education and self-realization | 1 | 0.1 |
| Education | 53 | Education and thinking | 1 | 0.1 |
| Education | 54 | Education, Intellect and Finances | 1 | 0.1 |
| Education | 55 | Education, career and income | 1 | 0.1 |
| Career | 56 | Employment | 31 | 2.4 |
| Other aspects | 57 | Everything | 5 | 0.4 |
| Other aspects | 58 | Everything I have is from parents | 1 | 0.1 |
| Family and relationships | 59 | Family | 9 | 0.7 |
| Other aspects | 60 | Fighting spirit | 1 | 0.1 |
| Income | 61 | Finances, Income | 1 | 0.1 |
| Income | 62 | Finances, by profession | 1 | 0.1 |
| Income | 63 | Finances, health | 1 | 0.1 |
| Income | 64 | Finances. education | 1 | 0.1 |
| Income | 65 | Financial stability | 7 | 0.6 |
| Other aspects | 66 | Freedom | 7 | 0.6 |
| Family and relationships | 67 | Friends | 16 | 1.3 |
| Other aspects | 68 | Goaltending | 5 | 0.4 |
| Other aspects | 69 | Good and secured life | 1 | 0.1 |
| Other aspects | 70 | Hard working | 7 | 0.6 |
| Family and relationships | 71 | Having children | 2 | 0.2 |
| Other aspects | 72 | Health | 3 | 0.2 |
| Other aspects | 73 | Honesty | 3 | 0.2 |
| Living conditions | 74 | Housing | 21 | 1.7 |
| Other aspects | 75 | Humanity, culture and manners | 2 | 0.2 |
| Other aspects | 76 | I am healthier | 1 | 0.1 |
| Living conditions | 77 | I am living in the worst condition, rat | 2 | 0.2 |
| Education | 78 | I am more educated, I had a career and job | 1 | 0.1 |
| Other aspects | 79 | I am more successful | 1 | 0.1 |
| Other aspects | 80 | I am more successful | 4 | 0.3 |
| Don't know | 81 | I don't compare | 1 | 0.1 |
| Other aspects | 82 | I had more possibilities to, rather than my parents | 1 | 0.1 |
| Other aspects | 83 | I have better life | 2 | 0.2 |
| Education | 84 | I have higher education, but I work less | 1 | 0.1 |
| Other aspects | 85 | I have my father’s specialty | 1 | 0.1 |
| Living conditions | 86 | I have my own residence | 1 | 0.1 |
| Other aspects | 87 | I know what the cost of work is | 1 | 0.1 |
| Other aspects | 88 | I used my abilities rationally | 1 | 0.1 |
| Family and relationships | 89 | I was not lucky in family life | 1 | 0.1 |
| Other aspects | 90 | I'm also successful | 1 | 0.1 |
| Other aspects | 91 | In that period (USSR) people lived in a | 1 | 0.1 |
| Income | 92 | Income | 138 | 10.9 |
| Income | 93 | Income and Education | 1 | 0.1 |
| Income | 94 | Income, career | 2 | 0.2 |
| Income | 95 | Income, children | 1 | 0.1 |
| Income | 96 | Income, education | 1 | 0.1 |
| Income | 97 | Income, education, business opportunity | 1 | 0.1 |
| Income | 98 | Income, health | 2 | 0.2 |
| Income | 99 | Income, housing | 1 | 0.1 |
| Other aspects | 100 | Independence | 3 | 0.2 |
| Other aspects | 101 | Intellect | 4 | 0.3 |
| Other aspects | 102 | Intellect and Hard work | 1 | 0.1 |
| Career | 103 | Job | 13 | 1.0 |
| Living conditions | 104 | Level of living | 44 | 3.5 |
| Other aspects | 105 | Life and financially | 1 | 0.1 |
| Other aspects | 106 | Life aspirations | 1 | 0.1 |
| Living conditions | 107 | Life better conditions | 1 | 0.1 |
| Other aspects | 108 | Life is different | 1 | 0.1 |
| Other aspects | 109 | Life was better | 1 | 0.1 |
| Other aspects | 110 | Lifestyle | 1 | 0.1 |
| Living conditions | 111 | Living conditions | 9 | 0.7 |
| Other aspects | 112 | Modern lifestyle | 5 | 0.4 |
| Other aspects | 113 | More action | 1 | 0.1 |
| Family and relationships | 114 | More children | 1 | 0.1 |
| Other aspects | 115 | More freedom to develop | 1 | 0.1 |
| Other aspects | 116 | More perspectives | 9 | 0.7 |
| Other aspects | 117 | More resources for life development | 1 | 0.1 |
| Other aspects | 118 | More work than children | 1 | 0.1 |
| Other aspects | 119 | My father had a Position and so did I | 1 | 0.1 |
| Other aspects | 120 | My father was a farmer | 1 | 0.1 |
| Other aspects | 121 | My father was dead | 1 | 0.1 |
| Other aspects | 122 | My father was repressed and this had an | 1 | 0.1 |
| Other aspects | 123 | My parents started everything from zero | 1 | 0.1 |
| Other aspects | 124 | My parents were hard working | 1 | 0.1 |
| Other aspects | 125 | My parents were living better rather than I | 3 | 0.2 |
| Other aspects | 126 | My parents were living better rather than I | 1 | 0.1 |
| Other aspects | 127 | My parents were more successful | 1 | 0.1 |
| Other aspects | 128 | My parents were much more successful | 2 | 0.2 |
| Don't know | 129 | Nothing | 17 | 1.3 |
| Other aspects | 130 | Opportunities | 1 | 0.1 |
| Other aspects | 131 | Peace | 4 | 0.3 |
| Other aspects | 132 | Peace, Justice, income | 1 | 0.1 |
| Other aspects | 133 | Peaceful life | 3 | 0.2 |
| Family and relationships | 134 | Personal relationship | 32 | 2.5 |
| Other aspects | 135 | Perspective | 1 | 0.1 |
| Economic conditions | 136 | Poverty | 1 | 0.1 |
| Other aspects | 137 | Property | 3 | 0.2 |
| Family and relationships | 138 | Relationships | 19 | 1.5 |
| Other aspects | 139 | Relationships and attitudes have change | 1 | 0.1 |
| Other aspects | 140 | Same | 3 | 0.2 |
| Other aspects | 141 | Self-realization | 4 | 0.3 |
| Other aspects | 142 | Social factors | 3 | 0.2 |
| Other aspects | 143 | Social factors, Income | 1 | 0.1 |
| Family and relationships | 144 | Social life | 5 | 0.4 |
| Other aspects | 145 | Soviet Union | 1 | 0.1 |
| Other aspects | 146 | Stability | 4 | 0.3 |
| Other aspects | 147 | Stability, work, income | 1 | 0.1 |
| Living conditions | 148 | Standard of living | 1 | 0.1 |
| Other aspects | 149 | Status | 2 | 0.2 |
| Other aspects | 150 | Success | 5 | 0.4 |
| Other aspects | 151 | Technological advancement | 3 | 0.2 |
| Other aspects | 152 | That period (USSR) was better | 4 | 0.3 |
| Other aspects | 153 | That period (USSR) was much better | 1 | 0.1 |
| Other aspects | 154 | They did a lot of things, built a house | 1 | 0.1 |
| Other aspects | 155 | There are more opportunities now | 4 | 0.3 |
| Other aspects | 156 | There was no such tense situation | 1 | 0.1 |
| Other aspects | 157 | They did a lot things | 2 | 0.2 |
| Other aspects | 158 | They did not love themselves as much as | 1 | 0.1 |
| Other aspects | 159 | They died way earlier | 1 | 0.1 |
| Other aspects | 160 | They lived in an orderly state | 1 | 0.1 |
| Living conditions | 161 | They were living in better conditions | 1 | 0.1 |
| Other aspects | 162 | They were happier | 1 | 0.1 |
| Other aspects | 163 | They were healthier than I am | 1 | 0.1 |
| Other aspects | 164 | They were more secured | 1 | 0.1 |
| Other aspects | 165 | They were struggling more and working harder | 1 | 0.1 |
| Other aspects | 166 | Time | 5 | 0.4 |
| Other aspects | 167 | Time and conditions | 4 | 0.3 |
| Other aspects | 168 | Traditions | 2 | 0.2 |
| Other aspects | 169 | Way of life | 10 | 0.8 |
| Other aspects | 170 | We are refugees | 1 | 0.1 |
| Other aspects | 171 | We do not have employment related to education | 1 | 0.1 |
| Other aspects | 172 | With life, hardship | 1 | 0.1 |
| Other aspects | 173 | Life was better | 1 | 0.1 |
| Other aspects | 174 | Lucky | 1 | 0.1 |

*Source:* The Perceived Social Mobility dataset.

**Table S3.** Cramér's Vs estimates for estimates involving parental and own education and correlation coefficients for all other estimates used in the analysis

|  | Physical health | Mental health | Life satisfaction | Perceived social mobility | Age | Gender | Parental education | Own education | Household income | Labour market status | Settlement type | IDP status | Interview date |
| --- | --- | --- | --- | --- | --- | --- | --- | --- | --- | --- | --- | --- | --- |
| Physical health | 1.000 |  |  |  |  |  |  |  |  |  |  |  |  |
| P-value |  |  |  |  |  |  |  |  |  |  |  |  |  |
| Mental health | 0.5244* | 1.000 |  |  |  |  |  |  |  |  |  |  |  |
| P-value | 0.000 |  |  |  |  |  |  |  |  |  |  |  |  |
| Life satisfaction | 0.2965* | 0.2292* | 1.000 |  |  |  |  |  |  |  |  |  |  |
| P-value | 0.000 | 0.000 |  |  |  |  |  |  |  |  |  |  |  |
| Perceived social mob. | 0.1313* | 0.1318* | 0.2474* | 1.000 |  |  |  |  |  |  |  |  |  |
| P-value | 0.000 | 0.000 | 0.000 |  |  |  |  |  |  |  |  |  |  |
| Age | -0.4482* | -0.2292* | -0.2180* | -0.0277 | 1.000 |  |  |  |  |  |  |  |  |
| P-value | 0.000 | 0.000 | 0.000 | 0.3395 |  |  |  |  |  |  |  |  |  |
| Gender | 0.1612* | 0.0741* | -0.0245 | 0.0415 | -0.0767* | 1.000 |  |  |  |  |  |  |  |
| P-value | 0.000 | 0.008 | 0.389 | 0.1522 | 0.006 |  |  |  |  |  |  |  |  |
| Parental education | 0.1290* | 0.0947* | 0.1347* | 0.0716 | 0.3343* | 0.0938* | 1.000 |  |  |  |  |  |  |
| P-value | 0.000 | 0.001 | 0.000 | 0.108 | 0.000 | 0.011 |  |  |  |  |  |  |  |
| Own education | 0.1292* | 0.0745* | 0.1619* | 0.1372* | 0.2980* | 0.1227* | 0.3094* | 1.000 |  |  |  |  |  |
| P-value | 0.000 | 0.049 | 0.000 | 0.000 | 0.000 | 0.000 | 0.000 |  |  |  |  |  |  |
| Household income | 0.2545* | 0.1563* | 0.1806* | 0.0780* | -0.2787* | 0.1258* | 0.1232* | 0.1729* | 1.000 |  |  |  |  |
| P-value | 0.000 | 0.000 | 0.000 | 0.007 | 0.0000 | 0.000 | 0.000 | 0.000 |  |  |  |  |  |
| Labour market status | -0.2740* | -0.1460* | -0.1331* | -0.1099* | 0.2265* | -0.2597* | 0.1651* | 0.1720* | -0.1477* | 1.000 |  |  |  |
| P-value | 0.000 | 0.000 | 0.000 | 0.000 | 0.000 | 0.000 | 0.000 | 0.000 | 0.0000 |  |  |  |  |
| Settlement type | -0.0062 | -0.014 | 0.0018 | 0.0072 | 0.0023 | -0.0347 | 0.2081* | 0.1782* | 0.0562* | 0.0630* | 1.000 |  |  |
| P-value | 0.8251 | 0.616 | 0.9486 | 0.805 | 0.9357 | 0.216 | 0.000 | 0.000 | 0.045 | 0.0248 |  |  |  |
| IDP status | -0.0119 | -0.0196 | -0.0840* | -0.0723* | -0.0091 | 0.0135 | 0.0704 | 0.0553 | -0.0259 | 0.0279 | 0.0060 | 1.000 |  |
| P-value | 0.6728 | 0.485 | 0.0031 | 0.0125 | 0.7450 | 0.6295 | 0.101 | 0.274 | 0.3568 | 0.3203 | 0.8322 |  |  |
| Interview date | -0.0325 | -0.0571* | -0.0248 | 0.0165 | -0.0103 | -0.0083 | 0.0958* | 0.0835 | -0.0121 | 0.0074 | 0.0131 | -0.0076 | 1.000 |
| P-value | 0.2468 | 0.0418 | 0.3831 | 0.5692 | 0.7131 | 0.7663 | 0.031 | 0.185 | 0.6669 | 0.7911 | 0.6401 | 0.7860 |  |

*Source:* Own calculations based on the Perceived Social Mobility dataset. * p<0.05 or higher significance.

**Table S4.** Descriptive statistics for control variables

| Variables | Mean (SD) / proportions |
| --- | --- |
| Age (min-max 18-90) | 48.8 (16.2) |
| Gender |  |
| Female | 55.7 |
| Male | 44.3 |
| Parental education |  |
| Primary | 8.7 |
| Secondary | 32.4 |
| Vocational | 17.9 |
| Tertiary | 40.9 |
| Respondents’ education |  |
| Primary | 3.4 |
| Secondary | 30.0 |
| Vocational | 22.5 |
| Tertiary | 44.1 |
| Household income |  |
| Bottom tertile | 28.1 |
| Middle tertile | 17.6 |
| Top tertile | 17.9 |
| Missing | 36.4 |
| Labor market status |  |
| Employed | 31.8 |
| Self-employed | 13.4 |
| Unemployed | 20.6 |
| Retired | 19.9 |
| Other | 14.2 |
| Settlement |  |
| Urban | 29.1 |
| Small town | 3.7 |
| Rural | 30.9 |
| Capital | 36.4 |
| IDP status |  |
| No | 92.8 |
| Yes | 7.2 |
| Interview date |  |
| 21 January 2021 | 10.3 |
| 22 January 2021 | 16.1 |
| 23 January 2021 | 16.4 |
| 24 January 2021 | 19.7 |
| 25 January 2021 | 17.9 |
| 26 January 2021 | 18.1 |
| 27 January 2021 | 1.2 |
| 28 January 2021 | 0.3 |

*Source:* Own calculations based on the Perceived Social Mobility dataset.

**Table S5.** The most important factors which individuals consider while making intergenerational comparisons by perceived social mobility

|  | Strongly downward | Downward | Immobile | Upward | Strongly upward | Total | N |
| --- | --- | --- | --- | --- | --- | --- | --- |
| Education | 11.8 | 10.5 | 9.7 | 19.5 | 19.0 | 13.8 | 160 |
| Income | 15.8 | 19.7 | 9.7 | 7.4 | 17.7 | 13.4 | 155 |
| Career | 15.8 | 11.9 | 7.6 | 13.7 | 12.7 | 11.9 | 138 |
| Economic | 14.5 | 11.7 | 2.1 | 6.3 | 13.9 | 8.4 | 97 |
| Living | 7.9 | 10.2 | 6.7 | 7.4 | 2.5 | 7.9 | 92 |
| Family | 9.2 | 9.0 | 12.2 | 8.8 | 6.3 | 9.4 | 109 |
| Don’t know | 13.2 | 7.0 | 37.0 | 14.8 | 17.7 | 16.7 | 194 |
| Other | 11.8 | 20.2 | 15.1 | 22.0 | 10.1 | 18.5 | 214 |
| Total | 100.0 | 100.0 | 100.0 | 100.0 | 100.0 | 100.0 | 1,159 |

*Notes:* Pearson chi^2^ test p-value=0.000, Kendall's tau-b test p-value=0.024. *Source:* Own calculations based on the Perceived Social Mobility dataset.

**Table S6.** Multinomial regression model of specific comparison categories, other category being the reference, relative risk ratios

|  | Education | Income | Career | Economic | Living | Family | Don’t know |
| --- | --- | --- | --- | --- | --- | --- | --- |
| *Perceived mobility (ref. immobile)* |  |  |  |  |  |  |  |
| Strongly downward | 1.75 | 2.1 | 2.45 | 8.52** | 1.52 | 0.94 | 0.47 |
|  | [0.60,5.11] | [0.76,5.78] | [0.87,6.91] | [2.34,30.94] | [0.46,5.01] | [0.31,2.84] | [0.18,1.26] |
| Downward | 0.87 | 1.55 | 1.09 | 4.26** | 1.19 | 0.54 | 0.15*** |
|  | [0.45,1.66] | [0.84,2.86] | [0.55,2.13] | [1.56,11.64] | [0.59,2.41] | [0.29,1.02] | [0.08,0.26] |
| Upward | 1.34 | 0.54 | 1.23 | 2.22 | 0.8 | 0.49* | 0.28*** |
|  | [0.72,2.48] | [0.27,1.07] | [0.63,2.40] | [0.78,6.32] | [0.39,1.68] | [0.26,0.94] | [0.17,0.47] |
| Strongly upward | 2.82* | 2.76 | 2.6 | 10.42*** | 0.57 | 0.78 | 0.71 |
|  | [1.03,7.74] | [1.00,7.63] | [0.87,7.76] | [2.81,38.61] | [0.11,3.00] | [0.23,2.65] | [0.27,1.84] |
| Sociodemographic variables |  |  |  |  |  |  |  |
| Age | 0.89** | 1.02 | 1.13* | 1.09 | 1.05 | 1.02 | 0.98 |
|  | [0.83,0.96] | [0.94,1.11] | [1.03,1.25] | [0.98,1.21] | [0.95,1.17] | [0.93,1.12] | [0.91,1.06] |
| Age squared | 1.00** | 1.00 | 1.00** | 1 | 1 | 1 | 1 |
|  | [1.00,1.00] | [1.00,1.00] | [1.00,1.00] | [1.00,1.00] | [1.00,1.00] | [1.00,1.00] | [1.00,1.00] |
| Male | 0.98 | 1.16 | 0.91 | 1.02 | 1.49 | 0.98 | 1.19 |
|  | [0.64,1.49] | [0.76,1.78] | [0.59,1.41] | [0.62,1.68] | [0.91,2.45] | [0.61,1.57] | [0.79,1.79] |
| AIC | 4602 |  |  |  |  |  |  |
| BIC | 4885.1 |  |  |  |  |  |  |
| Observations | 1159 |  |  |  |  |  |  |
| Pseudo R^2^ | 0.083 |  |  |  |  |  |  |

*Notes:* * p<0.05, ** p<0.01, *** p<0.001. 95% confidence intervals in brackets. All models also account for interview day fixed effects. *Source:* Own calculations based on the Perceived Social Mobility dataset.

**Table S7.** Perceived social mobility and wellbeing outcomes

|  | *Physical health*  *(1=very bad, very good=5)* | | *Mental health*  *(1=very bad, very good=5)* | | *Life satisfaction*  *(0=dissatisfied, 10=satisfied)* | |
| --- | --- | --- | --- | --- | --- | --- |
|  | Model 1: OLS without controls | Model 2: OLS  with controls | Model 3: OLS without controls | Model 4: OLS  with controls | Model 5: OLS without controls | Model 6: OLS  with controls |
| *Perceived mobility* |  |  |  |  |  |  |
| (Strongly) downward | -0.12 | -0.11 | -0.07 | -0.08 | -0.62*** | -0.52** |
|  | [-0.25,0.02] | [-0.23,0.00] | [-0.18,0.03] | [-0.19,0.03] | [-0.95,-0.28] | [-0.84,-0.21] |
| Immobile | Reference | Reference | Reference | Reference | Reference | Reference |
| (Strongly) upward | 0.19** | 0.05 | 0.12* | 0.05 | 0.79*** | 0.57*** |
|  | [0.05,0.33] | [-0.07,0.17] | [0.00,0.23] | [-0.07,0.16] | [0.46,1.12] | [0.24,0.89] |
| Intercept | 3.45*** | 4.60*** | 3.93*** | 4.26*** | 5.70*** | 5.82*** |
|  | [3.35,3.56] | [3.99,5.20] | [3.84,4.01] | [3.71,4.81] | [5.44,5.95] | [4.30,7.35] |
| AIC | 2871.32 | 2570.69 | 2508.65 | 2435.69 | 4773.22 | 4671.29 |
| BIC | 2886.36 | 2720.97 | 2523.69 | 2585.98 | 4788.19 | 4820.83 |
| Observations | 1159 | 1159 | 1159 | 1159 | 1159 | 1159 |
| Adjusted R^2^ | 0.02 | 0.26 | 0.01 | 0.07 | 0.07 | 0.16 |

*Notes:* * p<0.05, ** p<0.01, *** p<0.001. 95% confidence intervals in brackets. Models with control variables account for age, age squared, gender, interview day fixed effects, parental education, respondents’ education, household income, labor market status, type of settlement, IDP status. *Source:* Own calculations based on Perceived Social Mobility dataset.

**Table S8.** Variance inflation factors for all variables included in the analysis

|  | *Physical health*  *(1=very bad, very good=5)* | | *Mental health*  *(1=very bad, very good=5)* | | *Life satisfaction*  *(0=dissatisfied, 10=satisfied)* | |
| --- | --- | --- | --- | --- | --- | --- |
|  | VIF | 1/VIF | VIF | 1/VIF | VIF | 1/VIF |
| *Perceived mobility (ref. immobile)* |  |  |  |  |  |  |
| Downward | 1.75 | 0.57 | 1.75 | 0.57 | 1.76 | 0.57 |
| Upward | 1.74 | 0.57 | 1.74 | 0.57 | 1.76 | 0.57 |
| Age | 2.02 | 0.50 | 2.02 | 0.50 | 1.98 | 0.50 |
| Male | 1.24 | 0.81 | 1.24 | 0.81 | 1.24 | 0.81 |
| *Parental education* | 1.57 | 0.64 | 1.57 | 0.64 | 1.57 | 0.64 |
| *Respondents’ education* | 1.53 | 0.65 | 1.53 | 0.65 | 1.54 | 0.65 |
| *Income (ref. 1^st^ tertile)* |  |  |  |  |  |  |
| 2^nd^ tertile | 1.48 | 0.68 | 1.48 | 0.68 | 1.48 | 0.67 |
| 3^rd^ tertile | 1.74 | 0.58 | 1.74 | 0.58 | 1.74 | 0.58 |
| Missing | 1.69 | 0.59 | 1.69 | 0.59 | 1.69 | 0.59 |
| *Labour market (ref. employed)* |  |  |  |  |  |  |
| Self-employed | 1.33 | 0.75 | 1.33 | 0.75 | 1.33 | 0.75 |
| Unemployed | 1.51 | 0.66 | 1.51 | 0.66 | 1.5 | 0.67 |
| Retired | 2.24 | 0.45 | 2.24 | 0.45 | 2.21 | 0.45 |
| Other status | 1.49 | 0.67 | 1.49 | 0.67 | 1.49 | 0.67 |
| *Settlement (ref. urban)* |  |  |  |  |  |  |
| Small town | 1.12 | 0.90 | 1.12 | 0.90 | 1.11 | 0.90 |
| Rural | 1.53 | 0.65 | 1.53 | 0.65 | 1.53 | 0.65 |
| Capital | 1.53 | 0.65 | 1.53 | 0.65 | 1.53 | 0.65 |
| IDP status | 1.02 | 0.98 | 1.02 | 0.98 | 1.02 | 0.98 |
| Interview date *(ref. 20 January 2021)* |  |  |  |  |  |  |
| 21 January 2021 | 2.35 | 0.43 | 2.35 | 0.43 | 2.39 | 0.42 |
| 22 January 2021 | 2.35 | 0.43 | 2.35 | 0.43 | 2.4 | 0.42 |
| 23 January 2021 | 2.56 | 0.39 | 2.56 | 0.39 | 2.61 | 0.38 |
| 24 January 2021 | 2.43 | 0.41 | 2.43 | 0.41 | 2.46 | 0.41 |
| 25 January 2021 | 2.37 | 0.42 | 2.37 | 0.42 | 2.4 | 0.42 |
| 26 January 2021 | 1.12 | 0.89 | 1.12 | 0.89 | 1.13 | 0.89 |
| 27 January 2021 | 1.03 | 0.97 | 1.03 | 0.97 | 1.03 | 0.97 |
| Mean VIF | 1.70 |  | 1.70 |  | 1.70 |  |

*Source:* Own calculations based on Perceived Social Mobility dataset.

**Table S9.** Perceived social mobility and wellbeing outcomes using ordered probit regressions

|  | *Physical health*  *(1=very bad, very good=5)* | | *Mental health*  *(1=very bad, very good=5)* | |
| --- | --- | --- | --- | --- |
|  | Model 1: oprobit without controls | Model 2: oprobit  with controls | Model 3: oprobit without controls | Model 4: oprobit  with controls |
| *Perceived mobility* |  |  |  |  |
| (Strongly) downward | -0.21** | -0.15 | -0.15 | -0.12 |
|  | [-0.37,-0.05] | [-0.32,0.01] | [-0.31,0.01] | [-0.28,0.05] |
| Immobile | Reference | Reference | Reference | Reference |
| (Strongly) upward | 0.15 | 0.08 | 0.13 | 0.09 |
|  | [-0.01,0.32] | [-0.09,0.26] | [-0.04,0.31] | [-0.09,0.26] |
| Cutting points |  |  |  |  |
| Point 1 | -3.91*** | -4.31*** | -3.30*** | -3.35*** |
|  | [-4.63,-3.19] | [-5.24,-3.38] | [-3.99,-2.62] | [-4.20,-2.50] |
| Point 2 | -2.77*** | -3.14*** | -2.57*** | -2.57*** |
|  | [-3.46,-2.08] | [-4.05,-2.24] | [-3.27,-1.88] | [-3.43,-1.71] |
| Point 3 | -1.45*** | -1.75*** | -1.42*** | -1.39** |
|  | [-2.14,-0.77] | [-2.64,-0.86] | [-2.09,-0.75] | [-2.24,-0.55] |
| Point 4 | -0.01 | -0.26 | 0.22 | 0.27 |
|  | [-0.68,0.66] | [-1.13,0.62] | [-0.45,0.89] | [-0.57,1.11] |
| AIC | 2612.03 | 2555.69 | 2382.10 | 2374.98 |
| BIC | 2692.27 | 2721.00 | 2462.33 | 2540.29 |
| Observations | 1159 | 1159 | 1159 | 1159 |
| Pseudo R^2^ | 0.10 | 0.13 | 0.04 | 0.05 |

*Notes:* * p<0.05, ** p<0.01, *** p<0.001. 95% confidence intervals in brackets. All models account for age, age

squared, gender, and interview day fixed effects. Models with control variables account for age, age

squared, gender, interview day fixed effects, parental education, respondents’ education, household income,

labor market status, type of settlement, IDP status. *Source:* Own calculations based on Perceived Social Mobility dataset.

**Table S10.** Perceived social mobility and health and wellbeing outcomes

|  | *Physical health*  *(1=very bad, very good=5)* | | *Mental health*  *(1=very bad, very good=5)* | | *Life satisfaction*  *(0=dissatisfied, 10=satisfied)* | |
| --- | --- | --- | --- | --- | --- | --- |
|  | Model 1: OLS without controls | Model 2: OLS  with controls | Model 3: OLS without controls | Model 4: OLS without controls | Model 5: OLS  with controls | Model 6: OLS without controls |
| Intercept | 3.45*** | 4.60*** | 3.93*** | 4.26*** | 5.70*** | 5.82*** |
|  | [3.35,3.56] | [3.99,5.20] | [3.84,4.01] | [3.71,4.81] | [5.44,5.95] | [4.30,7.35] |
| *Perceived mobility (ref. immobile)* |  |  |  |  |  |  |
| Downward | -0.12 | -0.11 | -0.07 | -0.08 | -0.62*** | -0.52** |
|  | [-0.25,0.02] | [-0.23,0.00] | [-0.18,0.03] | [-0.19,0.03] | [-0.95,-0.28] | [-0.84,-0.21] |
| Upward | 0.19** | 0.05 | 0.12* | 0.05 | 0.79*** | 0.57*** |
|  | [0.05,0.33] | [-0.07,0.17] | [0.00,0.23] | [-0.07,0.16] | [0.46,1.12] | [0.24,0.89] |
| Age | –––––– | -0.04** | –––––– | -0.00 | –––––– | -0.05 |
|  | –––––– | [-0.06,-0.01] | –––––– | [-0.02,0.01] | –––––– | [-0.10,0.00] |
| Age squared | –––––– | 0.00 | –––––– | -0.00 | –––––– | 0.00 |
|  | –––––– | [-0.00,0.00] | –––––– | [-0.00,0.00] | –––––– | [-0.00,0.00] |
| Male | –––––– | 0.21*** | –––––– | 0.10* | –––––– | -0.13 |
|  | –––––– | [0.10,0.31] | –––––– | [0.01,0.20] | –––––– | [-0.40,0.14] |
| *Parental education (ref. primary)* |  |  |  |  |  |  |
| Secondary | –––––– | 0.08 | –––––– | 0.05 | –––––– | 0.20 |
|  | –––––– | [-0.12,0.28] | –––––– | [-0.12,0.21] | –––––– | [-0.32,0.72] |
| Vocational | –––––– | -0.03 | –––––– | -0.08 | –––––– | -0.07 |
|  | –––––– | [-0.25,0.19] | –––––– | [-0.27,0.10] | –––––– | [-0.64,0.50] |
| Tertiary | –––––– | 0.10 | –––––– | 0.03 | –––––– | 0.22 |
|  | –––––– | [-0.11,0.31] | –––––– | [-0.16,0.21] | –––––– | [-0.35,0.79] |
| *Respondents’ education (ref. primary)* |  |  |  |  |  |  |
| Secondary | –––––– | -0.02 | –––––– | -0.09 | –––––– | 0.74 |
|  | –––––– | [-0.37,0.33] | –––––– | [-0.39,0.21] | –––––– | [-0.07,1.55] |
| Vocational | –––––– | -0.02 | –––––– | 0.00 | –––––– | 0.76 |
|  | –––––– | [-0.38,0.34] | –––––– | [-0.30,0.31] | –––––– | [-0.07,1.59] |
| Tertiary | –––––– | 0.12 | –––––– | 0.04 | –––––– | 1.26** |
|  | –––––– | [-0.24,0.48] | –––––– | [-0.27,0.36] | –––––– | [0.41,2.10] |
| *Income (ref. 1^st^ tertile)* |  |  |  |  |  |  |
| 2^nd^ tertile | –––––– | 0.03 | –––––– | 0.15* | –––––– | 0.46* |
|  | –––––– | [-0.11,0.17] | –––––– | [0.02,0.27] | –––––– | [0.08,0.85] |
| 3^rd^ tertile | –––––– | 0.12 | –––––– | 0.09 | –––––– | 1.02*** |
|  | –––––– | [-0.04,0.28] | –––––– | [-0.06,0.24] | –––––– | [0.60,1.43] |
| Missing | –––––– | 0.16* | –––––– | 0.12* | –––––– | 0.49** |
|  | –––––– | [0.04,0.29] | –––––– | [0.00,0.24] | –––––– | [0.14,0.84] |
| *Labour market (ref. employed)* |  |  |  |  |  |  |
| Self-employed | –––––– | -0.05 | –––––– | -0.01 | –––––– | -0.13 |
|  | –––––– | [-0.21,0.11] | –––––– | [-0.17,0.14] | –––––– | [-0.57,0.30] |
| Unemployed | –––––– | -0.27*** | –––––– | -0.13* | –––––– | -0.58** |
|  | –––––– | [-0.41,-0.13] | –––––– | [-0.26,-0.00] | –––––– | [-0.95,-0.20] |
| Retired | –––––– | -0.42*** | –––––– | -0.03 | –––––– | -0.09 |
|  | –––––– | [-0.62,-0.23] | –––––– | [-0.21,0.14] | –––––– | [-0.62,0.43] |
| Other status | –––––– | -0.20** | –––––– | -0.06 | –––––– | 0.14 |
|  | –––––– | [-0.34,-0.05] | –––––– | [-0.21,0.08] | –––––– | [-0.30,0.58] |
| *Settlement (ref. urban)* |  |  |  |  |  |  |
| Small town | –––––– | 0.10 | –––––– | -0.02 | –––––– | 0.15 |
|  | –––––– | [-0.15,0.36] | –––––– | [-0.27,0.24] | –––––– | [-0.47,0.76] |
| Rural | –––––– | -0.09 | –––––– | -0.02 | –––––– | 0.02 |
|  | –––––– | [-0.21,0.03] | –––––– | [-0.14,0.09] | –––––– | [-0.33,0.36] |
| Capital | –––––– | 0.01 | –––––– | -0.04 | –––––– | -0.11 |
|  | –––––– | [-0.11,0.12] | –––––– | [-0.15,0.06] | –––––– | [-0.43,0.21] |
| IDP status | –––––– | -0.05 | –––––– | -0.02 | –––––– | -0.53* |
|  | –––––– | [-0.21,0.12] | –––––– | [-0.19,0.15] | –––––– | [-1.01,-0.05] |
| Interview date (20 January 2021) |  |  |  |  |  |  |
| 21 January 2021 | 0.12 | [-0.07,0.31] | 0.03 | [-0.15,0.21] | 0.20 | [-0.32,0.71] |
| 22 January 2021 | 0.10 | [-0.10,0.31] | 0.13 | [-0.05,0.30] | 0.24 | [-0.27,0.74] |
| 23 January 2021 | 0.05 | [-0.14,0.24] | 0.04 | [-0.14,0.21] | 0.26 | [-0.23,0.74] |
| 24 January 2021 | 0.05 | [-0.14,0.24] | -0.00 | [-0.17,0.17] | 0.01 | [-0.49,0.51] |
| 25 January 2021 | -0.07 | [-0.27,0.12] | -0.08 | [-0.25,0.09] | -0.08 | [-0.61,0.45] |
| 26 January 2021 | 0.42 | [-0.04,0.87] | -0.11 | [-0.88,0.65] | 0.48 | [-1.50,2.45] |
| 27 January 2021 | 0.33 | [-0.06,0.73] | 0.27 | [-0.12,0.66] | 2.24 | [-1.77,6.25] |
| AIC | 2871.32 | 2570.69 | 2508.65 | 2435.69 | 4773.22 | 4671.29 |
| BIC | 2886.36 | 2720.97 | 2523.69 | 2585.98 | 4788.19 | 4820.83 |
| Observations | 1159 | 1159 | 1159 | 1159 | 1159 | 1159 |
| Adjusted R^2^ | 0.02 | 0.26 | 0.01 | 0.07 | 0.07 | 0.16 |

*Notes:* * p<0.05, ** p<0.01, *** p<0.001. 95% confidence intervals in brackets. All models account for interview day fixed effects. *Source:*

Own calculations based on Perceived Social Mobility dataset.

**Table S11.** Perceived social mobility by comparison areas and health and wellbeing outcomes, without controls

|  | Model 1: OLS *Physical health*  *(1=very bad, very good=5)* | Model 2: OLS *Mental health*  *(1=very bad, very good=5)* | Model 3: OLS  *Life satisfaction*  *(0=dissatisfied, 10=satisfied)* |
| --- | --- | --- | --- |
| Intercept | 3.45*** | 3.93*** | 5.70*** |
|  | [3.35,3.56] | [3.84,4.01] | [5.44,5.96] |
| *Perceived mobility (ref. immobile)* |  |  |  |
| Downward: education | 0.23 | 0.05 | -0.14 |
|  | [-0.06,0.52] | [-0.19,0.29] | [-0.87,0.60] |
| Downward: Income | -0.31** | -0.21* | -1.20*** |
|  | [-0.52,-0.10] | [-0.41,-0.01] | [-1.71,-0.69] |
| Downward: Career | -0.05 | -0.19* | -0.45 |
|  | [-0.25,0.15] | [-0.39,-0.00] | [-1.03,0.14] |
| Downward: Economic | 0.13 | 0.23* | -0.75* |
|  | [-0.13,0.40] | [0.01,0.45] | [-1.43,-0.07] |
| Downward: Living | -0.22 | -0.20 | -0.76* |
|  | [-0.48,0.04] | [-0.42,0.01] | [-1.48,-0.05] |
| Downward: Family | -0.15 | -0.18 | -0.39 |
|  | [-0.44,0.14] | [-0.44,0.08] | [-1.18,0.39] |
| Downward: Don't know | -0.19 | -0.01 | -0.47 |
|  | [-0.48,0.10] | [-0.25,0.24] | [-1.14,0.20] |
| Downward: Other | -0.20 | -0.02 | -0.26 |
|  | [-0.43,0.03] | [-0.19,0.15] | [-0.86,0.33] |
| Upward: education | 0.19 | 0.10 | 1.09*** |
|  | [-0.04,0.43] | [-0.10,0.30] | [0.56,1.62] |
| Upward: Income | 0.18 | 0.30* | 0.92 |
|  | [-0.17,0.52] | [0.06,0.53] | [-0.07,1.91] |
| Upward: Career | 0.33** | 0.13 | 0.76* |
|  | [0.08,0.57] | [-0.07,0.33] | [0.13,1.40] |
| Upward: Economic | 0.20 | 0.25 | 0.61 |
|  | [-0.15,0.55] | [-0.08,0.57] | [-0.20,1.42] |
| Upward: Living | -0.12 | -0.11 | -0.03 |
|  | [-0.48,0.24] | [-0.40,0.18] | [-0.77,0.71] |
| Upward: Family | -0.02 | 0.01 | 0.52 |
|  | [-0.37,0.34] | [-0.20,0.22] | [-0.26,1.31] |
| Upward: Don't know | 0.19 | 0.13 | 0.52 |
|  | [-0.05,0.44] | [-0.11,0.37] | [-0.06,1.11] |
| Upward: Other | 0.27* | 0.12 | 1.18*** |
|  | [0.05,0.49] | [-0.08,0.33] | [0.66,1.70] |
| AIC | 2821.08 | 2465.35 | 4667.47 |
| BIC | 2905.96 | 2550.23 | 4751.93 |
| Observations | 1159 | 1159 | 1159 |
| Adjusted R^2^ | 0.03 | 0.02 | 0.08 |

*Notes:* * p<0.05, ** p<0.01, *** p<0.001. 95% confidence intervals in brackets. *Source:* Own calculations based on Perceived Social Mobility dataset.

**Table S12.** Perceived social mobility by comparison areas and health and wellbeing outcomes

|  | Model 1: OLS *Physical health*  *(1=very bad, very good=5)* | Model 2: OLS *Mental health*  *(1=very bad, very good=5)* | Model 3: OLS  *Life satisfaction*  *(0=dissatisfied, 10=satisfied)* |
| --- | --- | --- | --- |
| Intercept | 4.58*** | 4.29*** | 5.80*** |
|  | [3.96,5.21] | [3.74,4.84] | [4.22,7.38] |
| *Perceived mobility (ref. immobile)* |  |  |  |
| Downward: education | 0.02 | -0.04 | -0.42 |
|  | [-0.25,0.30] | [-0.28,0.19] | [-1.15,0.30] |
| Downward: Income | -0.21* | -0.15 | -0.87** |
|  | [-0.40,-0.02] | [-0.33,0.04] | [-1.39,-0.34] |
| Downward: Career | -0.15 | -0.27** | -0.57* |
|  | [-0.34,0.03] | [-0.46,-0.07] | [-1.11,-0.03] |
| Downward: Economic | 0.17 | 0.23* | -0.58 |
|  | [-0.05,0.40] | [0.00,0.45] | [-1.22,0.05] |
| Downward: Living | -0.21 | -0.20 | -0.63 |
|  | [-0.42,0.01] | [-0.40,0.01] | [-1.32,0.07] |
| Downward: Family | -0.17 | -0.19 | -0.40 |
|  | [-0.45,0.10] | [-0.45,0.07] | [-1.12,0.32] |
| Downward: Don't know | -0.14 | -0.01 | -0.18 |
|  | [-0.38,0.10] | [-0.25,0.23] | [-0.81,0.45] |
| Downward: Other | -0.14 | 0.00 | -0.13 |
|  | [-0.35,0.06] | [-0.17,0.17] | [-0.70,0.45] |
| Upward: education | 0.08 | 0.03 | 0.70** |
|  | [-0.13,0.30] | [-0.17,0.22] | [0.19,1.21] |
| Upward: Income | 0.18 | 0.28* | 1.02* |
|  | [-0.13,0.48] | [0.06,0.51] | [0.06,1.97] |
| Upward: Career | 0.03 | -0.02 | 0.35 |
|  | [-0.17,0.24] | [-0.23,0.18] | [-0.28,0.99] |
| Upward: Economic | 0.25 | 0.25 | 0.56 |
|  | [-0.04,0.54] | [-0.06,0.56] | [-0.26,1.38] |
| Upward: Living | -0.12 | -0.11 | -0.00 |
|  | [-0.43,0.19] | [-0.38,0.16] | [-0.73,0.73] |
| Upward: Family | -0.16 | -0.06 | 0.46 |
|  | [-0.45,0.13] | [-0.27,0.15] | [-0.35,1.27] |
| Upward: Don't know | 0.10 | 0.08 | 0.42 |
|  | [-0.13,0.34] | [-0.16,0.32] | [-0.16,1.00] |
| Upward: Other | 0.04 | 0.02 | 0.88** |
|  | [-0.17,0.25] | [-0.19,0.22] | [0.34,1.41] |
| Age | -0.03** | -0.01 | -0.05 |
|  | [-0.06,-0.01] | [-0.03,0.01] | [-0.10,0.00] |
| Age squared | 0.00 | -0.00 | 0.00 |
|  | [-0.00,0.00] | [-0.00,0.00] | [-0.00,0.00] |
| Male | 0.20*** | 0.10 | -0.11 |
|  | [0.10,0.31] | [-0.00,0.19] | [-0.39,0.17] |
| *Parental education (ref. primary)* |  |  |  |
| Secondary | 0.08 | 0.02 | 0.18 |
|  | [-0.13,0.28] | [-0.15,0.19] | [-0.34,0.69] |
| Vocational | -0.03 | -0.10 | -0.09 |
|  | [-0.25,0.20] | [-0.30,0.09] | [-0.66,0.48] |
| Tertiary | 0.08 | -0.00 | 0.15 |
|  | [-0.14,0.30] | [-0.19,0.19] | [-0.42,0.72] |
| *Respondents’ education (ref. primary)* |  |  |  |
| Secondary | -0.01 | -0.05 | 0.86* |
|  | [-0.36,0.34] | [-0.34,0.24] | [0.04,1.68] |
| Vocational | -0.03 | 0.02 | 0.87* |
|  | [-0.38,0.33] | [-0.28,0.31] | [0.03,1.72] |
| Tertiary | 0.13 | 0.09 | 1.35** |
|  | [-0.23,0.48] | [-0.22,0.39] | [0.49,2.21] |
| *Income (ref. 1^st^ tertile)* |  |  |  |
| 2^nd^ tertile | 0.02 | 0.15* | 0.50* |
|  | [-0.12,0.16] | [0.02,0.28] | [0.11,0.89] |
| 3^rd^ tertile | 0.10 | 0.07 | 0.98*** |
|  | [-0.06,0.26] | [-0.08,0.22] | [0.56,1.41] |
| Missing | 0.15* | 0.11 | 0.53** |
|  | [0.02,0.28] | [-0.01,0.23] | [0.18,0.87] |
| *Labour market (ref. employed)* |  |  |  |
| Self-employed | -0.08 | -0.04 | -0.11 |
|  | [-0.24,0.09] | [-0.19,0.12] | [-0.55,0.33] |
| Unemployed | -0.27*** | -0.13 | -0.59** |
|  | [-0.42,-0.12] | [-0.26,0.00] | [-0.97,-0.20] |
| Retired | -0.44*** | -0.09 | -0.20 |
|  | [-0.64,-0.24] | [-0.27,0.09] | [-0.74,0.34] |
| Other status | -0.20** | -0.05 | 0.15 |
|  | [-0.35,-0.05] | [-0.20,0.10] | [-0.30,0.60] |
| *Settlement (ref. urban)* |  |  |  |
| Small town | 0.14 | 0.01 | 0.14 |
|  | [-0.11,0.40] | [-0.25,0.26] | [-0.48,0.77] |
| Rural | -0.09 | -0.02 | 0.04 |
|  | [-0.21,0.04] | [-0.14,0.09] | [-0.31,0.38] |
| Capital | 0.01 | -0.03 | -0.10 |
|  | [-0.11,0.13] | [-0.14,0.07] | [-0.42,0.23] |
| IDP status | -0.05 | -0.02 | -0.54* |
|  | [-0.22,0.12] | [-0.19,0.15] | [-1.04,-0.05] |
| AIC | 2535.88 | 2393.66 | 4579.48 |
| BIC | 2755.33 | 2613.11 | 4797.82 |
| Observations | 1159 | 1159 | 1159 |
| Adjusted R^2^ | 0.26 | 0.08 | 0.16 |

*Notes:* * p<0.05, ** p<0.01, *** p<0.001. 95% confidence intervals in brackets. All models also account for interview day fixed effects. *Source:* Own calculations based on Perceived Social Mobility dataset.

**Figure S1:** Kerned density estimate of life satisfaction variable

*Source:* Own calculations based on Perceived Social Mobility dataset.

**Figure S2.** Distribution of areas of intergenerational comparison.

*Source:* Own calculations based on the Perceived Social Mobility dataset.
